# Deep Learning-Based Spectrum Sensing for Cognitive Radio Applications

**DOI:** 10.3390/s24247907

**Published:** 2024-12-11

**Authors:** Sara E. Abdelbaset, Hossam M. Kasem, Ashraf A. Khalaf, Amr H. Hussein, Ahmed A. Kabeel

**Affiliations:** 1 Electronics and Electrical Communications Engineering Department, Higher Institute of Engineering and Technology, New Damietta 34517, Egypt; sara.abdelbaset@ndeti.edu.eg; 2 Electronics and Communications Department, Faculty of Engineering, Tanta University, Tanta 31511, Egypt or hossam.kasem@ejust.edu.eg (H.M.K.); amr.abdallah@f-eng.tanta.edu.eg (A.H.H.); 3 Computer Science Engineering, Egypt-Japan University of Science and Technology (E-JUST), Alexandria 21934, Egypt; 4 Electronics and Electrical Communication Department, Faculty of Engineering, AL Menya University, Minia 61519, Egypt; ashraf.khalaf@mu.edu.eg; 5 Electronics and Communication Department, Faculty of Engineering, Delta University for Science and Technology, Gamasa 35511, Egypt

**Keywords:** convolutional neural networks, deep learning, spectrum sensing, cognitive radio

## Abstract

In order for cognitive radios to identify and take advantage of unused frequency bands, spectrum sensing is essential. Conventional techniques for spectrum sensing rely on extracting features from received signals at specific locations. However, convolutional neural networks (CNNs) and recurrent neural networks (RNNs) have recently demonstrated promise in improving the precision and efficacy of spectrum sensing. Our research introduces a groundbreaking approach to spectrum sensing by leveraging convolutional neural networks (CNNs) to significantly advance the precision and effectiveness of identifying unused frequency bands. We treat spectrum sensing as a classification task and train our model with diverse signal types and noise data, enabling unparalleled adaptability to novel signals. Our method surpasses traditional techniques such as the maximum–minimum eigenvalue ratio-based and frequency domain entropy-based methods, showcasing superior performance and adaptability. In particular, our CNN-based approach demonstrates exceptional accuracy, even outperforming established methods when faced with additive white Gaussian noise (AWGN).

## 1. Introduction

There is a shortage of frequency spectrum as a result of growing network needs and the introduction of 6G applications like Industry 5.0, Intelligent Healthcare, and the Internet of Everything, which are driving up demand for radio resources [1,2]. Unlicensed secondary users (SUs) cannot access primary users’ (PUs) radio resources, even when they are not in use, because traditional static spectrum allocation systems only allow licensed PUs to use the allocated spectrum [3]. It is essential to fully utilize underutilized spectrum bands in order to prevent denying essential radio demands. A method of spectrum management called dynamic spectrum management (DSM) uses spectrum sensing (SS) to find the PUs’ idle frequency bands and allows SUs to send within them. False alarms (FAs) and missing PU detections are problems with traditional spectrum sensing techniques like energy detection and cyclostationary-based detection [4]. Researchers have looked into using typical machine learning (ML) algorithms, such as support vector machines and the naïve Bayes classifier, to enhance sensing performance in order to overcome these drawbacks of conventional spectrum sensing techniques. But conventional machine learning techniques usually require a time-consuming manual feature extraction procedure that depends on domain knowledge [5,6].

In [7], the author presents a novel and significant contribution to the field of cognitive radio networks (CRNs) and ambient backscatter communication. By integrating energy-harvesting capabilities with cooperative spectrum sensing and ambient backscatter assistance, the study aims to reduce the age of information (AoI) in wireless networks. The findings demonstrate the potential for significant advancements in minimizing (AoI) through the proposed framework. Leveraging cooperative spectrum sensing and ambient backscatter assistance in energy-harvesting CRNs represents a promising approach towards enhancing network efficiency and reducing latency.

Deep learning is employed within wireless networks for tasks such as spectrum sensing, signal modulation recognition, multiple-input-multiple-output systems, non-orthogonal multiple access, and resource allocation [8,9]. Using knowledge from previous radio spectrum allocation scenarios, deep learning-based dynamic spectrum sensing seeks to minimize errors in channel identification and classification. This method builds a sensing model by leveraging the learning powers of recurrent neural networks (RNNs) and convolutional neural networks (CNNs). RNNs take advantage of the temporal dynamics present in the spectrum, whereas CNNs are used for feature extraction [10,11,12]. This paper frames spectrum sensing as a central difficulty and attempts to construct a deep learning classification-based solution. The CNN uses the signal’s power spectrum as its input, and the network is trained using a variety of signal and noise datasets.

The goal of this research is to create a deep learning classification system, and the topic of spectrum sensing is introduced. The following are this article’s main contributions:

- The present approach has been compared with conventional techniques for spectrum sensing, such as the frequency domain entropy-based method and the maximum–minimum eigenvalue ratio-based method. Based on the same chance of false alarm, the results demonstrate that our method’s detection probability is higher than the that of the two standard approaches.
- Signal kinds that have not been trained have been used in experiments. As demonstrated by the results, the suggested approach is able to adjust to the identification of these unknown signals, demonstrating the high degree of generalizability of the model that our method produces.
- The method’s performance has been investigated under AWGN. The results show that the proposed deep learning-based method performs much better in terms of detection performance under AWGN than the conventional methods based on the maximum–minimum eigenvalue ratio and frequency domain entropy. This further confirms the superiority of our approach.

The rest of the paper is organized in the following manner. Section 2 lists related work. In Section 3, the spectrum sensing problem—which can be viewed as a two-category classification problem—is discussed. Section 4 gives a detailed description of the proposed deep neural network-based method. The results of the simulation are shown in Section 5, and the conclusions are provided in Section 6.

## 2. Related Work

Eigenvalue-based detection [11], frequency domain entropy-based detection [12], cyclostationary feature-based detection [13], matched filter [14], and the power spectral density split cancellation-based method [15] are all examples of traditional spectrum sensing techniques. The simplicity of energy detection makes it popular, but noise uncertainty is a problem. Recent advancements, such as energy detection across multiple alternating time slots, have been proposed to address this limitation, enhancing performance under dynamic conditions [16]. The detection of cyclostationary features, on the other hand, requires signal expertise and computational complexity, yet performs well in low signal-to-noise areas. Eigenvalues are used to make decisions in eigenvalue-based detection, which is resistant against noise uncertainty. Entropy-based methods in the frequency domain distinguish between signal and noise distributions. Sub-band power ratios are used in the power spectral density split cancellation method, which reduces noise effects but necessitates limited frequency monitoring bands [17,18,19,20].

The integration of machine learning into spectrum sensing has gained traction due to technological advancements, particularly in cooperative spectrum sensing. For instance, researchers have explored cooperative sensing algorithms leveraging Gaussian mixture models, K-means clustering, weighted K-closest neighbors, and support vector machines (SVM) [21,22,23,24,25,26] with received signal energy as the feature vector. Other studies have applied K-means clustering and SVM with low-dimensional probability vectors.

Additionally, AI-driven deep learning techniques are widely employed across various applications, and our current focus lies in exploring their application in spectrum sensing. This paper delves into several research endeavors centered around applying spectrum sensing to deep learning. An artificial neural network-based method for spectrum sensing was presented by Vyas et al. [27], who used likelihood ratio test statistics and signal energy as training features. A model based on CNNs was proposed by Han et al. [23] for cyclostationary feature detection and energy signal training. By using a CNN to improve sensing decisions among individual SUs via hard or soft combinations, Lee et al. [25] presented a deep cooperative spectrum sensing method that outperformed traditional methods in terms of accuracy. Similarly, capsule networks and cyclic cumulants have been employed in deep-learning-based spectrum sensing, demonstrating effective classification of modulated signals with high accuracy [28]. Using phase components for model training and evaluation across different channel types, Chandhok et al. [21] introduced ‘SenseNet’, a novel architecture for wideband spectrum sensing and automatic modulation classification. Zheng et al. [29] presented the sensing issue as a classification problem, addressing noise power uncertainties by training the model on received signal power, incorporating transfer learning for improved performance compared to existing methods. The effectiveness of a robust spectrum sensing approach that integrates transfer learning was validated by Peng et al. [30]. With covariance matrices as inputs, Xie et al. [31] presented a CNN-LSTM detector for spectrum sensing that demonstrated superiority in both noisy and noise-free circumstances. For orthogonal frequency-division multiplexing (OFDM) systems, Cheng et al. [32] presented a deep learning-based spectrum sensing technique that uses stacked auto-encoders to extract features. Using the structural information of modulated signals, Gao et al. [33] developed the ‘DetectNet’ and ‘SoftCombinationNet’ models for spectrum and cooperative spectrum sensing, much outperforming conventional techniques. Additionally, custom feature-extraction layers have been integrated into deep-learning-based classifiers, achieving an enhanced performance in digitally modulated signal detection [34]. These studies collectively demonstrate the superior performance of deep learning in spectrum sensing when compared to traditional methods, which inspired the research described in this paper and was motivated by the findings of Cheng et al. [35] and Gao et al. [36].

In particular, CNNs are generally employed in image processing, but they can also interpret the spatial properties of radio frequency (RF) signals and convert them into observable patterns [37]. On the other hand, RNNs are very good at analyzing sequences, which makes them perfect for monitoring the changes in spectrum consumption over time. When coupled, these algorithms have the ability to completely transform spectrum sensing, allowing for a more flexible and effective use of the radio frequency spectrum to maximize available spectrum resources and satisfy the demands of contemporary wireless communication. Energy detection, matched filtering, and cyclostationary feature detection are examples of traditional spectrum sensing techniques that face a number of difficulties. Energy detection, for example, is simple, but it suffers in settings with varying noise levels. Although it requires prior knowledge of the principal user’s signal, matched filtering improves reliability. An adaptive thresholding approach to average energy detection has been shown to mitigate these challenges, offering a more robust spectrum sensing solution in cognitive radio systems [38]. Detecting cyclostationary features requires a lot of processing resources, even with noise robustness. In order to determine spectrum occupancy by comparing received and expected pilot signals, pilot-based sensing uses periodic pilot signal transmissions to identify primary users and estimate channel characteristics. Statistical methods like Bayesian approaches and hypothesis testing are essential to spectrum sensing because they allow for well-informed choices on the presence of signals based on statistical models and observable data. For example, Bayesian methods use data updates and prior probabilities to evaluate the likelihood of a signal, whereas hypothesis testing entails creating null and alternative hypotheses and making a decision based on statistical significance [39,40,41,42].

## 3. Problem Formulation

The core of the spectrum sensing problem lies in distinguishing between the two states of the radio frequency spectrum—whether it is occupied by a primary user (PU) or not. This binary classification problem can be framed as a hypothesis testing problem, which is central to understanding and solving the spectrum sensing challenge.

### 3.1. Binary Hypothesis Testing Problem

In spectrum sensing, we can model the received signal ***r***(n) on the sensing device using two hypotheses:

- $H_{0}$: No PU activity (the spectrum is idle)

The received signal in this case is just noise ***w***(n), with no signal from the primary user (PU):

(1) $$r\left(n\right)=w\left(n\right)$$

- $H_{1}$: PU is transmitting (the spectrum is occupied)

Here, the received signal is composed of both the signal from the PU and noise w(n), affected by a channel gain h:

(2) $$r\left(n\right)=hs\left(n\right)+w\left(n\right)$$

where ***s***(n) is the signal transmitted by the PU, ***w***(n) is the noise, and h is the channel gain.

This binary hypothesis test is formulated as:

(3) $$H_{0}:r\left(n\right)=w\left(n\right)\;(\mathrm{No PU signal})$$

(4) $$H_{1}:r\left(n\right)=hs\left(n\right)+w\;(\mathrm{PU signal is present})$$

### 3.2. Spectrum Sensing as a Classification Problem

From a classification perspective, this problem can be viewed as determining whether the received signal corresponds to $H_{0}$ (only noise) or $H_{1}$ (signal plus noise). The classification decision is made based on the received signal, and a detection algorithm is used to decide which hypothesis ($H_{0}$ or $H_{1}$) is more likely.

Thus, this binary classification problem can be tackled using machine learning and deep learning techniques, which can learn patterns from previous data and generalize to new, unseen scenarios.

### 3.3. Probability of Detection ($P_{d}$) and Probability of False Alarm ($P_{FA}$)

The performance of spectrum sensing is evaluated by two important metrics:

- Probability of Detection ($P_{d}$): This is the probability that the spectrum sensing algorithm correctly detects the presence of the PU when the PU signal is indeed present, i.e., when *H1* is true. It is defined as:

(5) $$P_{d}=P(correctly\;detect\;PU{|H}_{1})$$

Mathematically, this is the probability of correctly deciding $H_{1}$, when the true state is $H_{1}$:

(6) $$P_{d}=P({Ĥ}_{1}=H_{1}{|H}_{1})$$

- Probability of False Alarm $\left(P_{FA}\right)$

This is the probability that the spectrum sensing algorithm incorrectly detects the presence of a PU when the spectrum is actually idle, i.e., when *H*_0_ is true.

(7) $$P_{FA}=P(incorrectly\;detect\;PU{|H}_{0})$$

Mathematically, this is the probability of incorrectly deciding $H_{1}$ when the true state is $H_{0}$:

(8) $$P_{FA}=P({Ĥ}_{1}=H_{1}{|H}_{0})$$

### 3.4. Derivation of $P_{d}$ and $\;P_{FA}$

To derive $P_{d}$ and $P_{FA}$, let us consider the decision rule based on a threshold *y*, where the received signal ***r***(n) is compared to the threshold to decide whether $H_{1}$ (PU is present) or $H_{0}$ (PU is absent) is more likely. The decision rule can be expressed as:

(9) $${Ĥ}_{1}=\left\{\begin{array}{c} H_{1},\;if\;r(n)\geq \gamma \\ H_{0},\;if\;r(n)<\gamma \end{array}\right.$$

- Probability of Detection ($P_{d}$):

For $P_{d}$, we want to calculate the probability of correctly deciding $H_{1}$ when the PU is transmitting (i.e., the true state is $H_{1}$). This involves calculating the likelihood that the received signal ***r***(n) = *h**s***(n) + ***w***(n) exceeds the threshold y under the condition that $H_{1}$ is true:

(10) $$P_{d}=P(r(n)\geq \gamma {|H}_{1})$$

Given the statistical properties of the signal and noise, this probability can be computed using the likelihood function of the received signal under hypothesis $H_{1}$.

- Probability of False Alarm ($P_{FA}$):

For $P_{FA}$, we want to calculate the probability that the spectrum sensing algorithm incorrectly detects the presence of a PU when the true state is $H_{0}$. This means the decision rule will incorrectly decide $H_{1}$ when the received signal ***r***(n) = ***w***(n) is just noise:

(11) $$P_{FA}=P({Ĥ}_{1}=H_{1}{|H}_{0})$$

Again, using the statistical properties of the noise distribution, this probability is calculated based on the distribution of the received signal under hypothesis $H_{0}$.

### 3.5. Threshold Selection and Trade-Off Between $P_{d}$ and $P_{FA}$

In practice, there is a trade-off between the probability of detection and the probability of false alarm. Increasing $P_{d}$ (improving the detection of the PU signal) often results in an increase in $P_{FA}$ (more false alarms), and vice versa. This trade-off can be controlled by adjusting the threshold *y*. The optimal threshold is typically chosen to balance these two probabilities based on the desired performance of the spectrum sensing system.

The relationship between $P_{d}$ and $P_{FA}$ can be visualized using a receiver operating characteristic (ROC) curve, which plots $P_{d}$ against $P_{FA}$ for various threshold values. The curve helps in selecting the threshold that provides the best compromise between detection accuracy and false alarm rate.

### 3.6. Deep Learning in Spectrum Sensing

In the context of deep learning, these probabilities are evaluated using the output of the deep neural network (e.g., CNN or RNN). The model learns to map the received signal to either $H_{0}$ or $H_{1}$, and based on the network’s output, the probabilities $P_{d}$ and $P_{FA}$ are computed. The deep learning model aims to optimize these probabilities by training on large datasets of labeled signal and noise samples.

The following equations summarize the performance evaluation:

(12) $$P_{d}=\frac{Number\;of\;correct\;detections}{Total\;number\;of\;actual\;PU\;transmissions}$$

(13) $$P_{FA}=\frac{Number\;of\;false\;alarms}{\begin{array}{c} Total\;number\;of\;actual\;idle\;spectrum\;periods \end{array}}$$

These metrics allow for a quantitative comparison between the proposed deep learning-based spectrum sensing technique and traditional methods, highlighting the improvements in terms of detection accuracy and robustness.

## 4. Proposed Deep Learning-Based Solution

Our suggested deep learning model for spectrum sensing is presented in this section. One popular deep learning architecture used widely in computer vision, a subfield of AI devoted to helping computers understand and analyze visual or image data, is the CNN [42,43,44,45,46,47]. In the realm of machine learning, artificial neural networks demonstrate exceptional performance. These networks find utility across diverse datasets encompassing images, audio, and text. Various types of neural networks serve specific functions; convolutional neural networks are preferred for tasks like picture classification, whereas recurrent neural networks—in particular, long short-term memory (LSTM) networks—are used for word sequence prediction. This blog aims to construct a fundamental component for a CNN. Ordinary neural networks consist of three primary types of layers:

(a) Input Layers: The model’s first input is received by this layer. The entire number of characteristics in the data (such as pixels in the case of images) is equal to the number of neurons in this layer.
(b) Hidden Layers: Data move into the hidden layers following input from the input layer. Depending on its complexity and the extent of the dataset, the model could include several hidden layers. The number of neurons in each buried layer can vary, but they usually outnumber the characteristics. Each layer’s output is obtained by multiplying the output of the layer before it by the learnable weights unique to that layer. The addition of learnable biases and activation functions, which give the network nonlinearity, comes next.
(c) Output Layer: A logistic function such as SoftMax or Sigmoid receives the output produced by the hidden layer. These functions convert each class’ output into its corresponding probability score.

### 4.1. The Training Method

**Figure 1:**
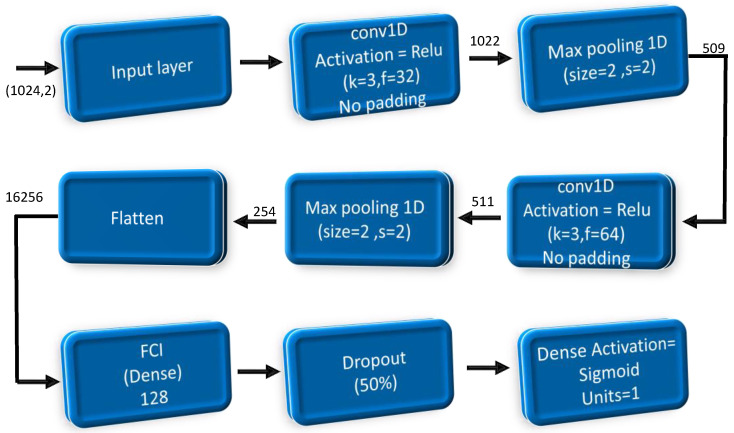
The proposed network flowchart.

Various types of wireless signals have been integrated into our training dataset to facilitate the recognition of diverse radio signals. This step aims to enable the trained network model to potentially identify unfamiliar signal types. The training dataset comprises noise data of equal size alongside the signal data. The signal, with a length of 1024 and two vectors, in-phase and quadrature components (I and Q), is processed in the following manner:

Initially, the signal is fed into a traditional one-dimensional (1D) layer with specifications: kernel size = 3, stride = 1, and 32 filters. No padding is required, resulting in a signal length of 1022. Subsequently, the signal, now 1022 in length, undergoes max pooling in a 1D layer with the following parameters: kernel size = 2 and stride = 2, reducing the signal length to 511. The 511-length signal is then passed through another 1D layer with kernel size = 3 and 64 filters, resulting in a signal size of 509. Following this, the signal is subjected to another max pooling 1D layer with kernel size = 2 and stride = 2, decreasing the signal length to 254. A neural network or a fully connected layer must process the signals in order to identify whether the input represents a signal or noise. However, with 64 filters and a length of 254 each, this setup is unsuitable for a fully connected layer. Therefore, the signal must first pass through a flattening layer without any trainable parameters. After flattening, the signal can enter a fully connected layer comprising 128 neural networks. Given the substantial number of connections, a dropout layer is introduced to regularize the model and prevent overfitting. Finally, a dense layer is employed to make the ultimate decision based on the processed signal. Our proposed network is shown in Figure 1.

Additionally, Table 1 shows the number of trained parameters for each layer included in the proposed network.

### 4.2. Content Loss

In this section, we suggest using the Binary Cross Entropy as a loss function in deep learning and machine learning to evaluate differences between predicted binary results and true binary labels. This metric evaluates the variance between probability distributions, enhancing model training by imposing penalties on erroneous predictions. The Binary Cross Entropy finds extensive application in tasks such as binary classification, which involves segregating data into two distinct classes.:

(14) $$logloss=-\frac{1}{n}\sum_{i}^{n}\sum_{j}^{M}y_{ij}log(p_{ij})$$

Here is the breakdown of the components of this formula:

- n the total number of samples or instances in the dataset.
- M the number of classes.

## 5. Experimental Result Analysis and Evaluations

We conducted a number of experiments to evaluate the performance of our suggested framework, and we presented the findings and analysis of our experiments. We tested the performance of the suggested network using a variety of experimental techniques, to make it practical for benchmarking and comparative investigations.

## 6. Experiment Design and Setup

### 6.1. Data Generation

The Radio ML 2018.01A dataset has been utilized, encompassing 24 modulation varieties such as QPSK, FM, GMSK, OOK, ASK4, ASK8, BPSK, QPSK, PSK8, PSK16, PSK32, APSK16, APSK32, APSK64, APSK128, QAM16, QAM32, QAM64, QAM128, QAM256, AM_SSB_WC, AM_SSB_SC, AM_DSB_WC, AM_DSB_SC, and more. A total of 26 signal-to-noise ratios (SNRs) ranging from −20 dB to +30 dB in 2 dB increments are used to represent each modulation type. Each modulation type contains 4096 frames per SNR, with each frame having a frame shape of 1024, 2, and 1024 complex time-series samples represented as floating-point in-phase and quadrature (I/Q) components. As a result, there are 2,555,904 frames in the collection. The dataset specification is displayed in Table 2.

### 6.2. Simulation Results

We use the stochastic gradient descent (SGD) optimization approach with a learning rate of 0.001 and no learning rate degradation to train our network. Momentum is set at 0 and weight decay to 0.0001. Additionally, our suggested framework undergoes end-to-end training. All experiments are trained on an NVIDIA Tesla P100 across 10 epochs with a batch size of 128. All experiments’ evaluation results are shown using metrics commonly used to assess performance, such as accuracy (prediction accuracy, probability of detection).

The training phase took place on Kaggle. By conducting an in-depth exploration of the Radio ML Dataset, we were able to leverage its coverage of 24 distinct modulation types. Detailed statistics were extracted for each modulation type, encompassing metrics like minimum, maximum, mean, and standard deviation. Subsequently, a comprehensive profile was developed for each modulation type, providing a holistic view of their characteristics.

First, for each modulation type, histograms were generated to visually represent the distribution of data. All 24 modulation types were employed in training a deep learning model aimed at distinguishing between signals and noise, thereby enabling the identification of the presence of PUs. To ensure robustness, the training encompassed scenarios representative of spectrum sensing in various systems, utilizing 26 different signal-to-noise ratios (SNRs) for each modulation type. Within the SNR context, 409 signals were employed, each comprising 1024 samples split into two frames, one of which represents the signal’s quadrature (Q) component and the other its in-phase (I) component.

A total of 255,591 signals and 255,591 noises were used for training, meaning that the total number of signals trained on is about 511,182 and the number of epochs equal 10.

**Figure 2:**
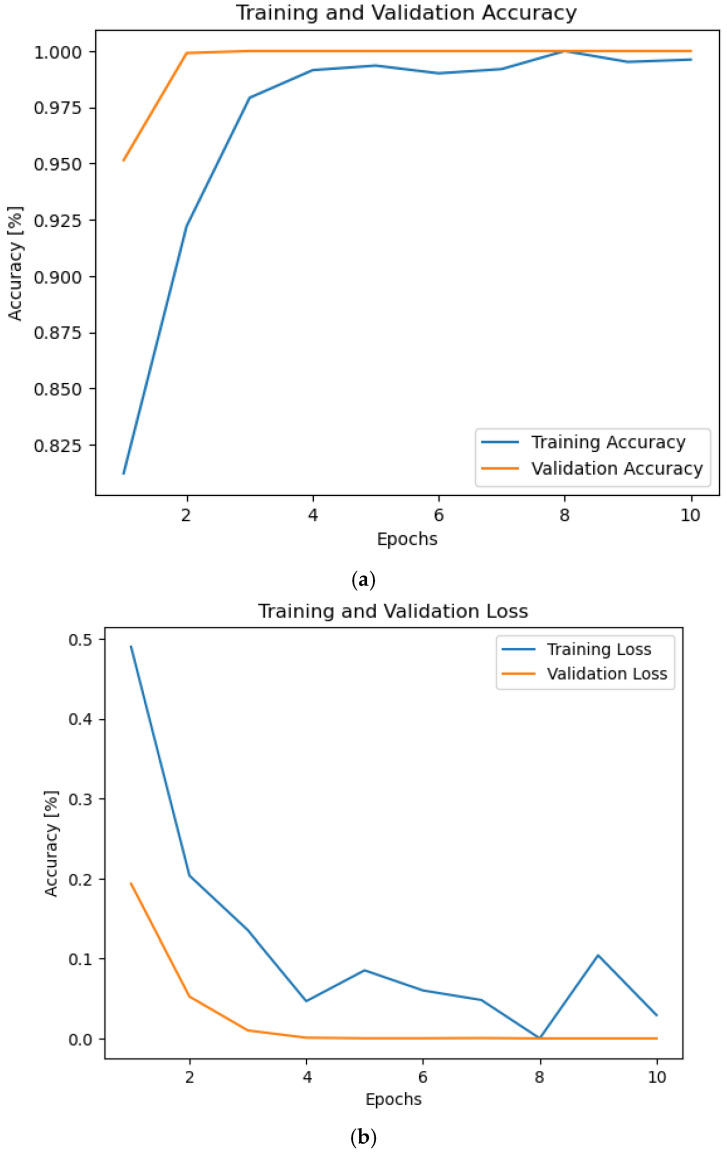
This graph shows the accuracy and losses for the number of epochs 10 during the training and validation phases. (**a**) Accuracy and (**b**) loss.

Without noise, the accuracy and losses during the training and validation stages are displayed in Figure 2. We can infer from Figure 2 that our suggested framework can achieve 100% at epoch 2. It means that the proposed framework can classify the different modulation types with minimum effort, using small number of training epochs.

**Figure 3:**
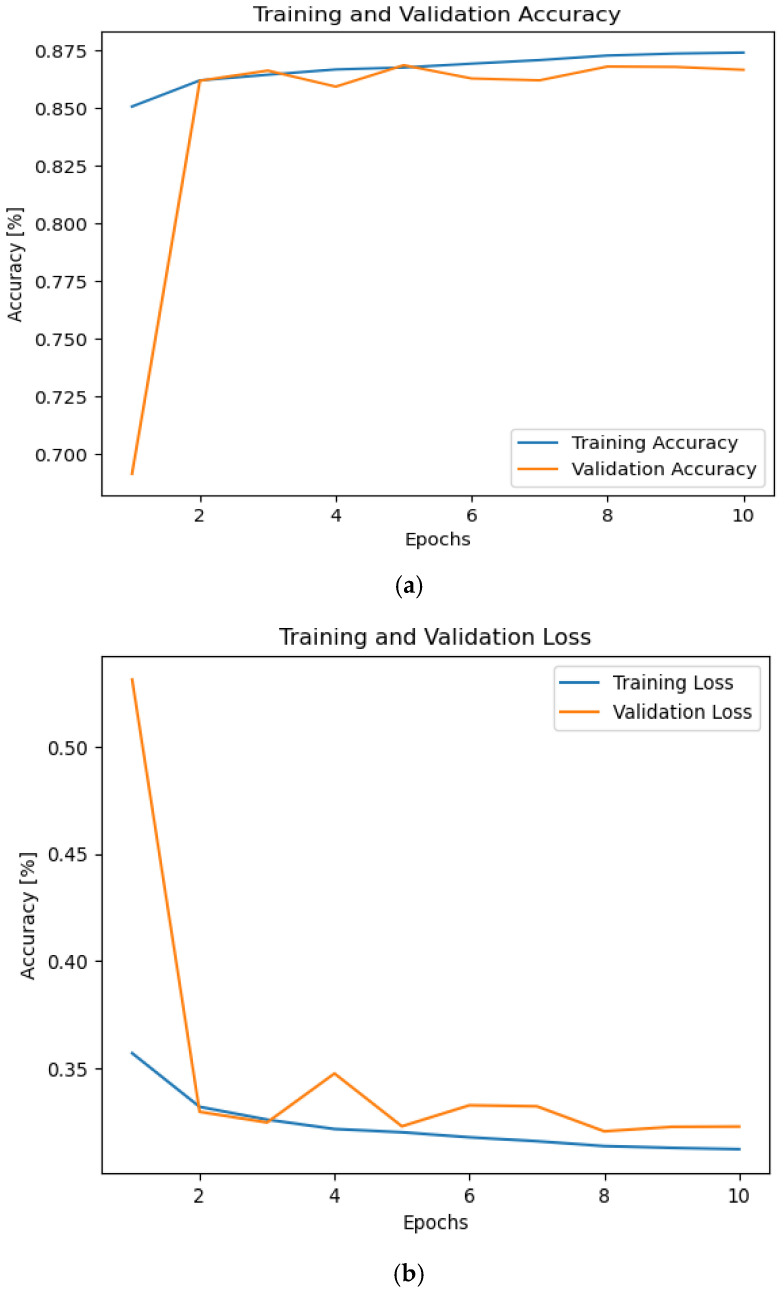
The accuracy and losses during training and validation phases with the effect of AWGN with No. of epochs 10. (**a**) Accuracy and (**b**) loss.

To test the proposed framework under noise effect, we generated AWGN and drew a histogram for one of the frames in it to ensure how close it was to the original signals, and the description of all the noises has been shown. The generated AWGN has a standard deviation of 0.708. Figure 3 shows the simulation results. As shown, the accuracy has been decreased to 87.5%. This is because of the effect of the noise of the proposed framework. Additionally, the proposed framework takes a much longer epoch to reach the minimum local mean.

**Figure 4:**
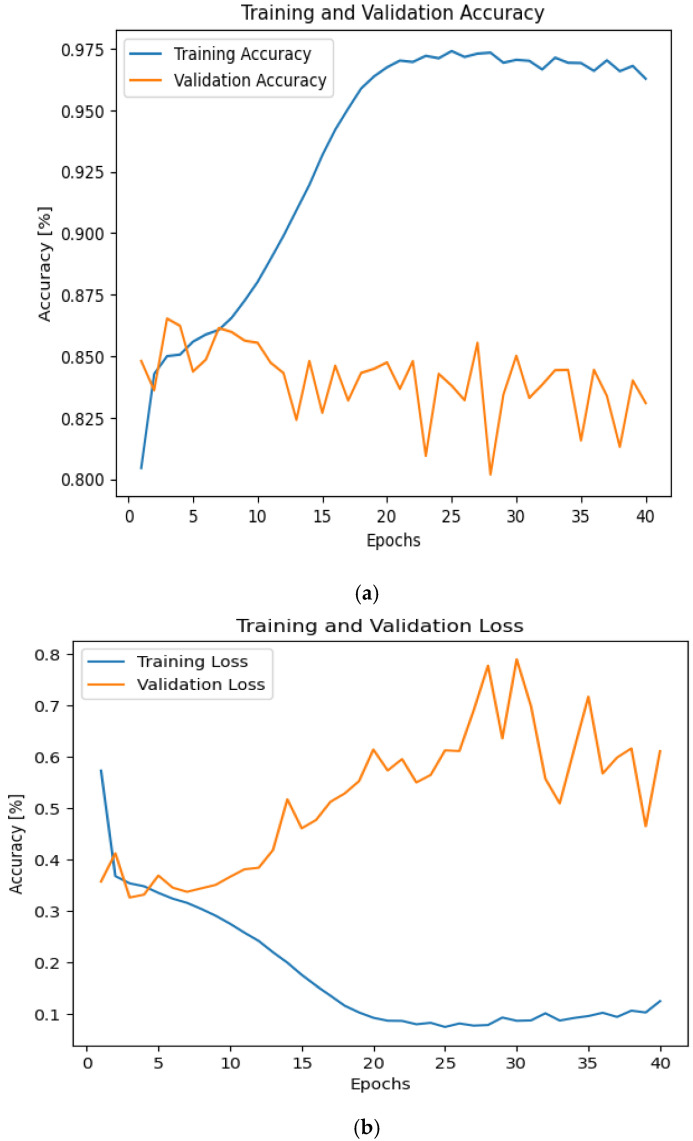
The accuracy and losses during training and validation phases with the effect of AWGN with No. of epochs 40. (**a**) Accuracy and (**b**) loss.

As shown in Figure 3, the accuracy fell to 87.5%. Therefore, we proposed to solve this challenge using various solutions. First, we suggest increasing the batch size to 2048 and the number of epochs to 40. Figure 4 displays the findings. Figure 4 shows us that the accuracy has increased to 97.5%. However, we noticed that an overfitting occurred in our proposed model.

As we noticed above, increasing No. of epoch and batch size leads to overfitting. Consequently, we changed the model structure to be as shown in Table 3. The simulation results of this network structure can reduce the overfitting that happened in the first network structure with the same accuracy value of 97.5%.

**Figure 5:**
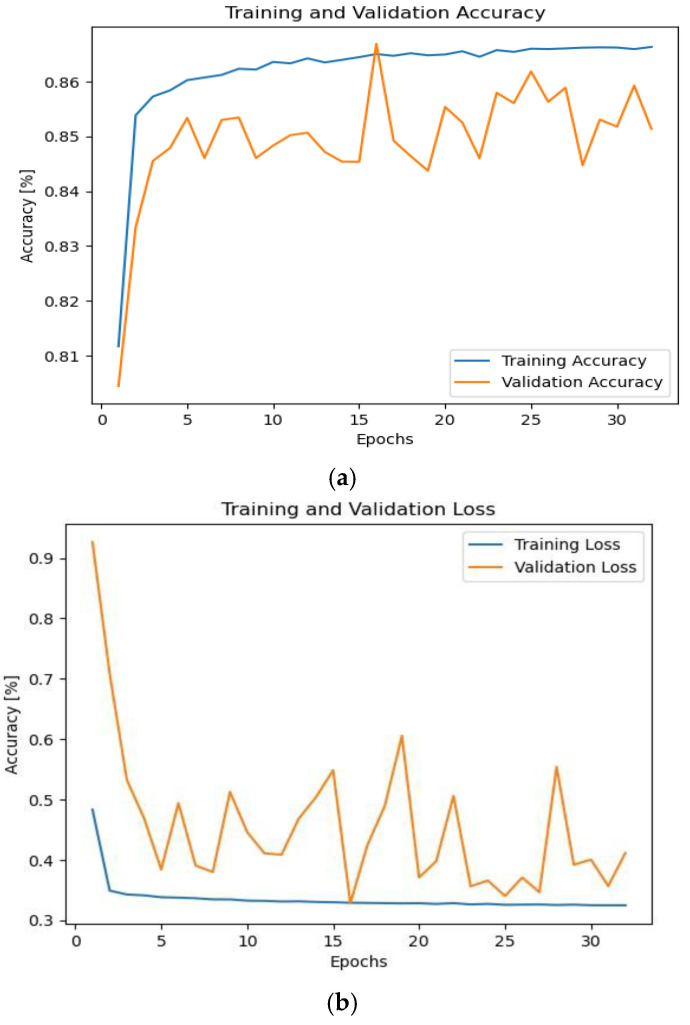
The accuracy and losses during training and validation phases with the effect of AWGN with SNR range (−8 to 30 dB) with No. of epochs 20. (**a**) Accuracy and (**b**) loss.

**Figure 6:**
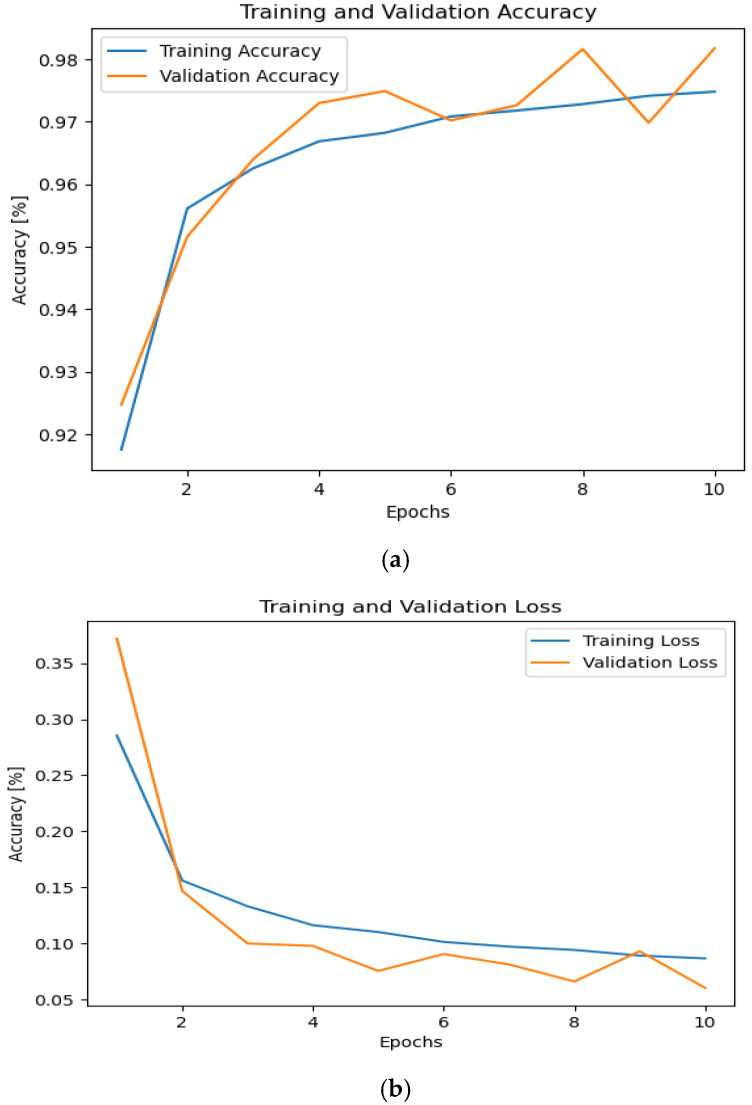
The accuracy and losses during training and validation phases with the effect of AWGN with SNR range (−8 to 30 dB) with No. of epochs 32. (**a**) Accuracy and (**b**) loss.

When the SNR distribution was examined more closely, it was observed that there were 10 SNRs that fell within the negative range, indicating a low signal strength relative to the noise level. As a result, the signal power was extremely low in comparison to the noise power. This means that we cannot detect the signal on this negative of SNR values. To validate this assumption, a histogram detailing all SNRs was constructed. When analyzing the modulation type orbit, it became apparent that the distribution of small SNRs closely resembled the noise samples used during training. The subsequent action involved excluding a segment of the small SNRs and retraining the model to ensure higher training accuracy. By utilizing only 20 SNRs (ranging from −8 to 30) instead of the initial 26 (ranging from −20 to 30) and training the model over 10 epochs, the accuracy surged to 98%, with a loss of less than 0.1%, as depicted in Figure 5. Subsequently, the model underwent training for 32 epochs to further enhance accuracy, surpassing 98% accuracy and maintaining a loss under 0.05% by incorporating a flattening layer, as illustrated in Figure 6.

**Figure 7:**
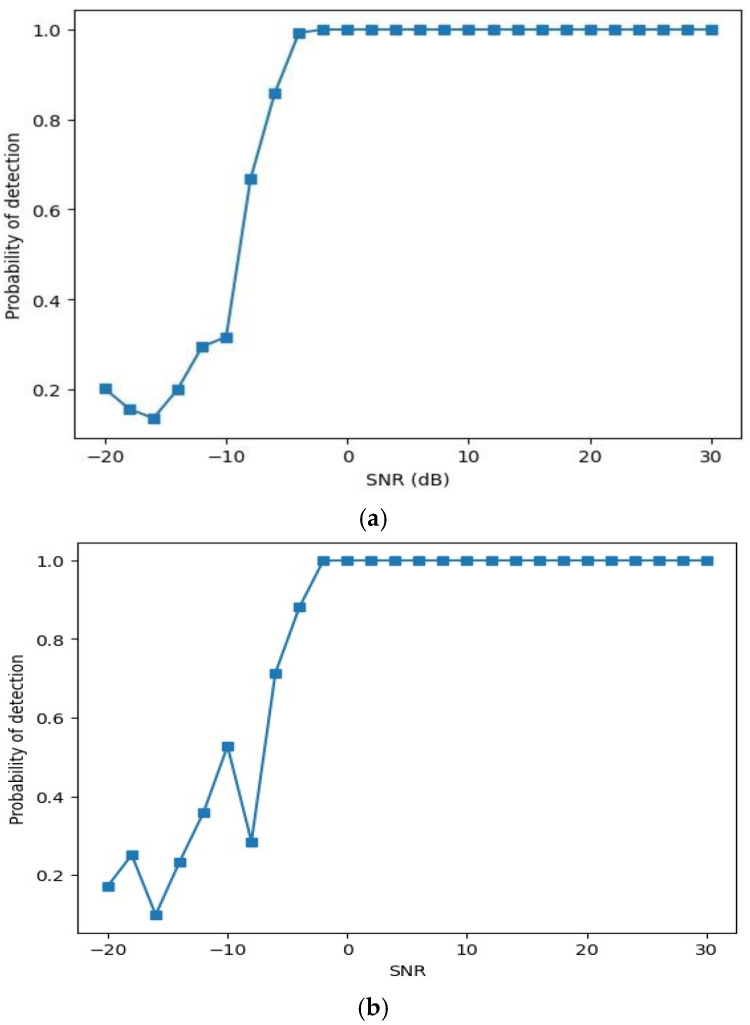
Detection performance (**a**) OOK, (**b**) QPSK.

In order to evaluate the model’s generalization across all SNRs, Figure 7 shows how SNR and the probability of detection (Pd) for on-off keying (OOK) and quadrature phase-shift keying (QPSK) modulations relate to whether a PU is present or not. Notably, when observing Pd values at low SNRs, they fall below 0.5, indicating the absence of a PU. As the SNR rises, Pd surpasses 0.5 and approaches 1, indicative of a presence of a PU. For instance, starting from −8, there is a slight increase in Pd. From −2 onwards, it consistently indicates the presence of a pure signal. This analysis helps in understanding how the model performs across varying SNR levels and modulation types, determining, from the signal characteristics, if a primary user is present or not.

### 6.3. Testing the Performance with Various Proposed Network Structures

**Figure 8:**
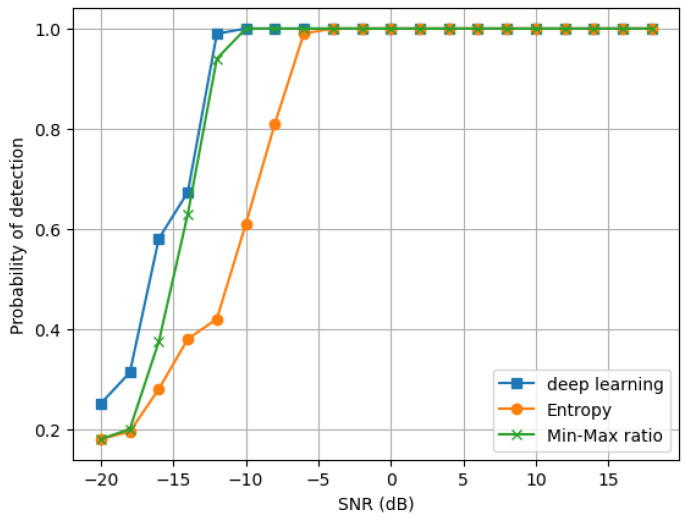
Comparison with traditional methods at pf = 0.15.

**Figure 9:**
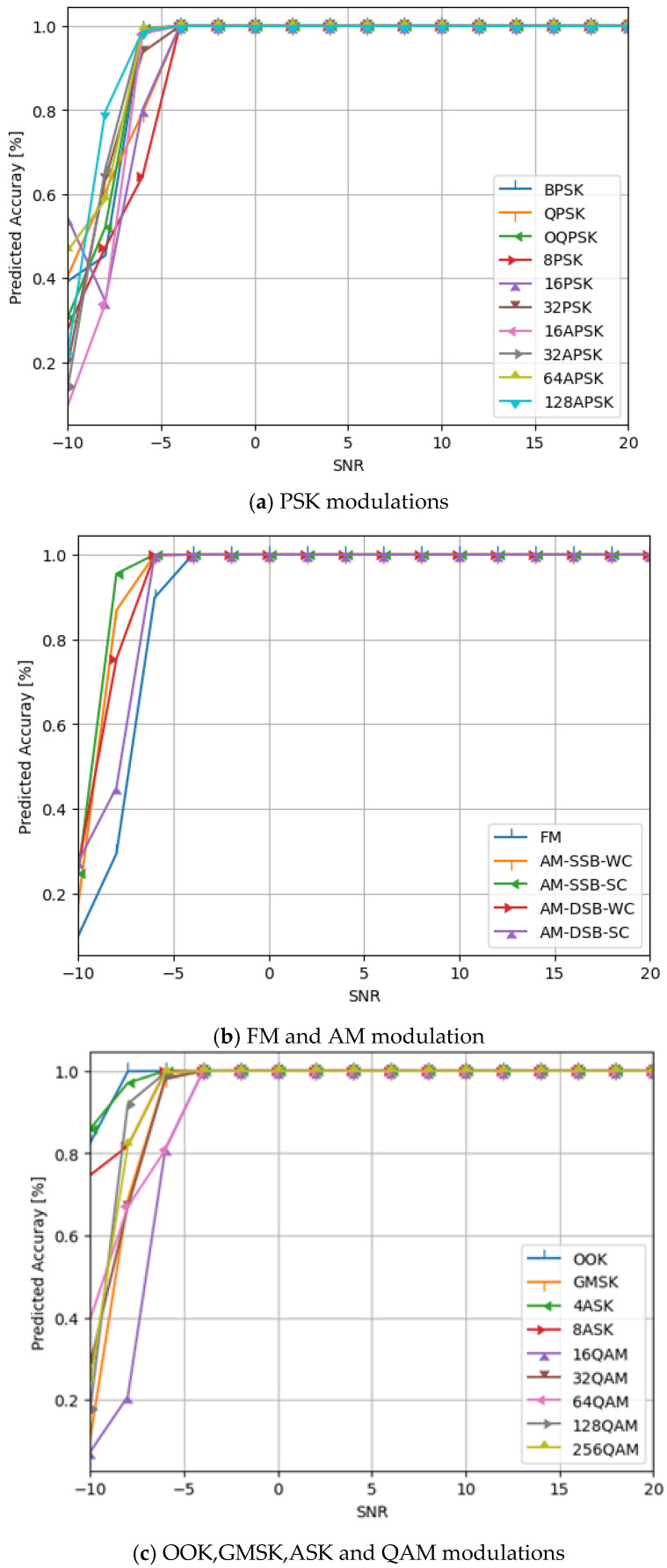
The predicted accuracy of our proposed model by using flatten layer for various modulation types.

Additionally, we evaluate the network structures’ performance as displayed in Table 1, with various modulation types included in the datasets against different SNRs values. Figure 8 displays the simulation findings. See also Figure 9.

**Figure 10:**
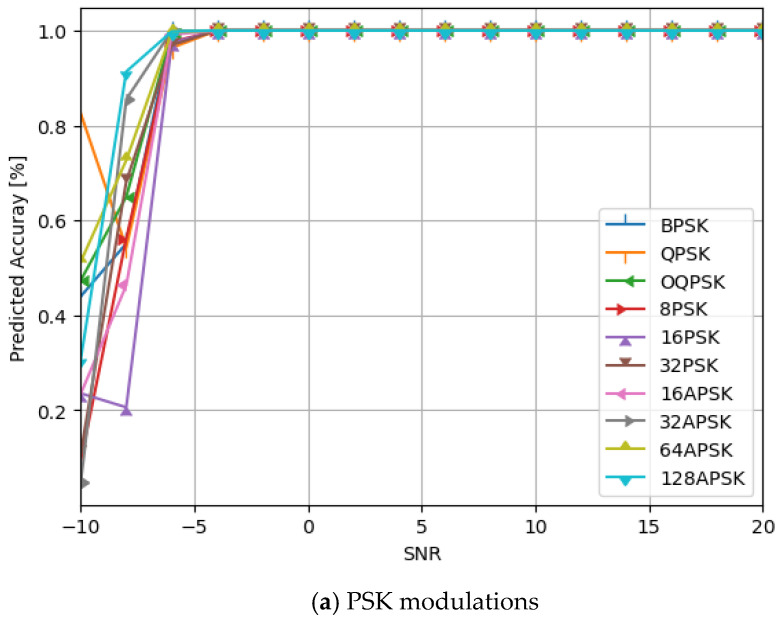

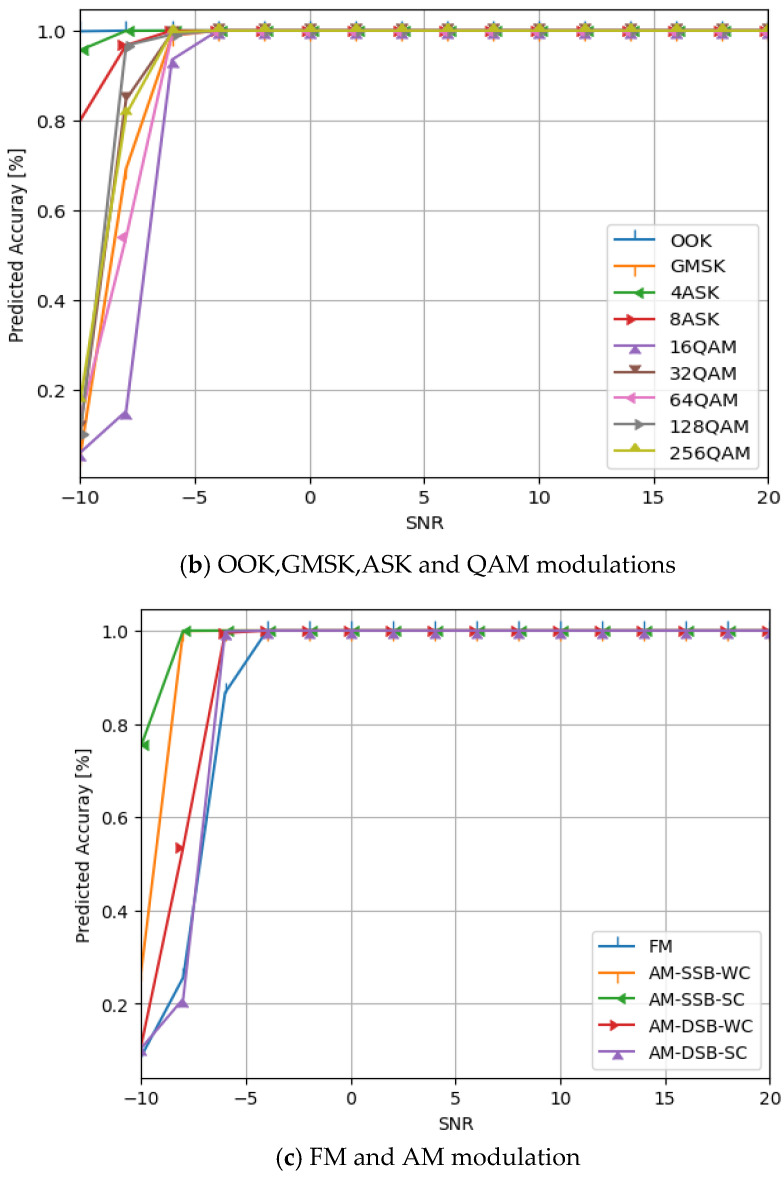
The predicted accuracy of our proposed mode by using GAP layer for various modulation types.

**Figure 11:**
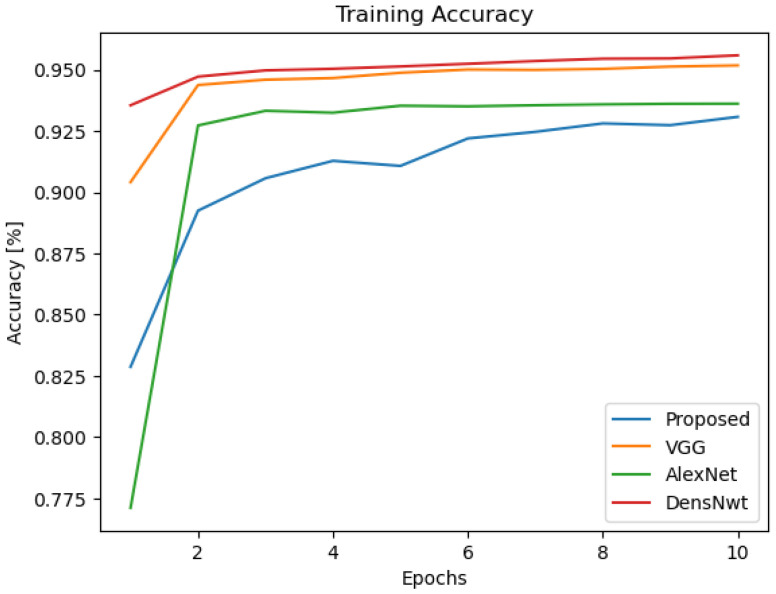
Training accuracy of the compared models.

**Figure 12:**
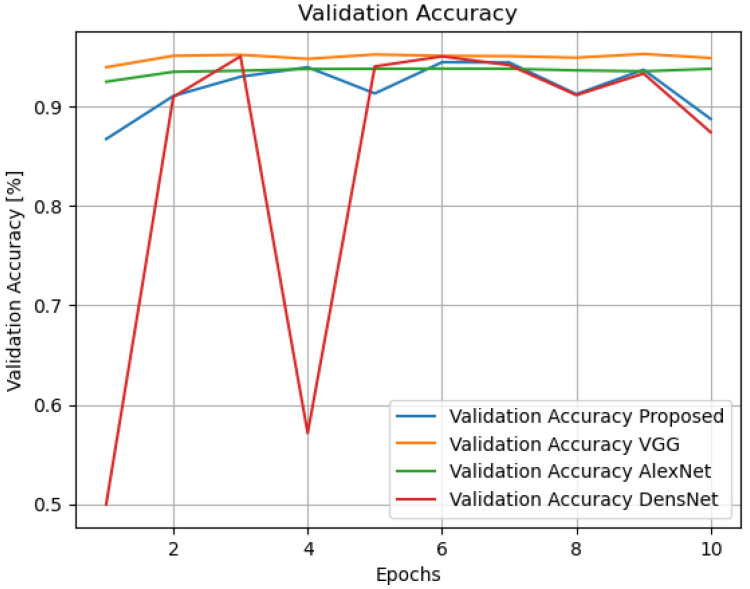
Validation accuracy of the compared models.

**Figure 13:**
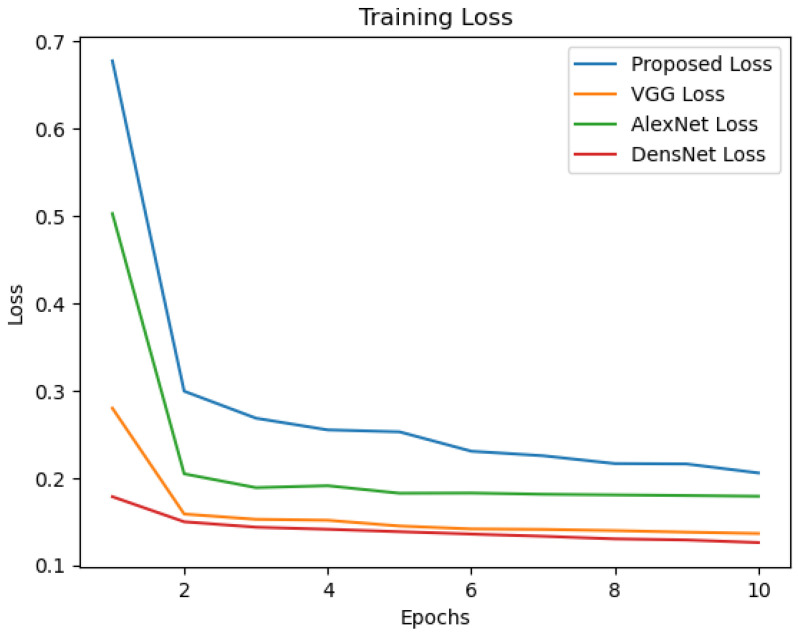
Training loss of the compared models.

**Figure 14:**
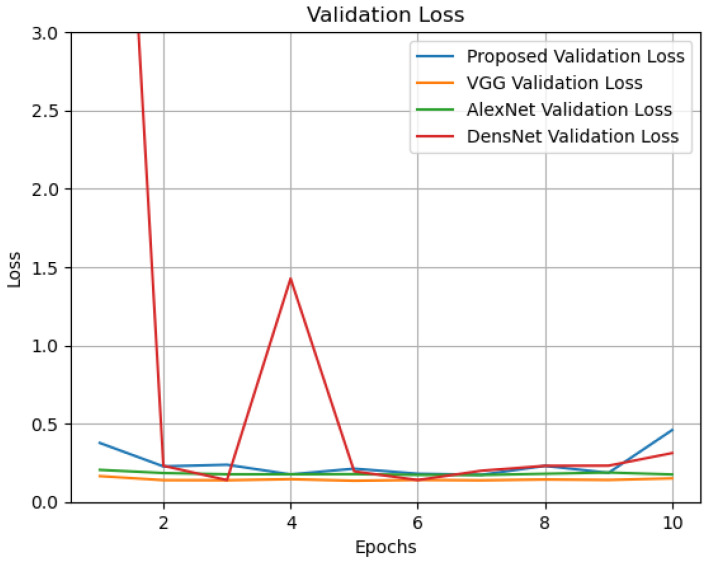
Validation loss of the compared models.

**Figure 15:**
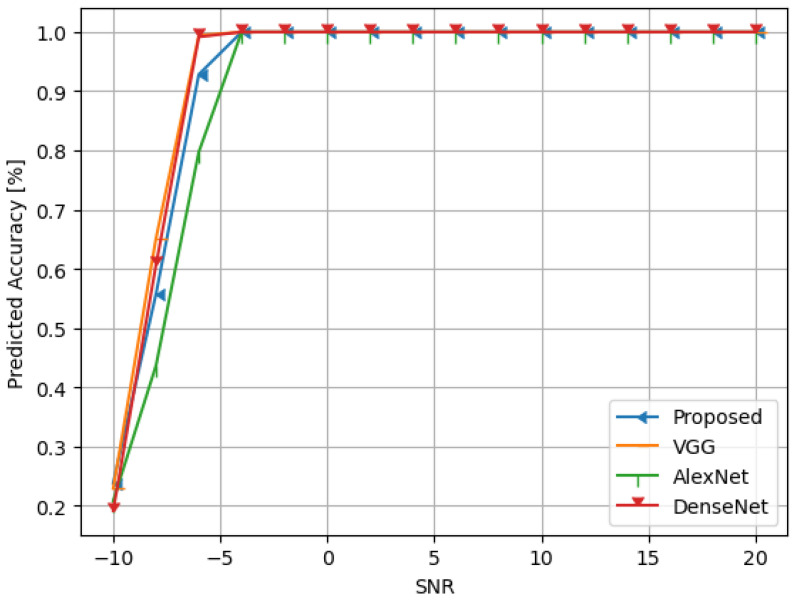
The predicted accuracy of the compared models at SNR from −10 to 20.

Additionally, as seen in Table 4, we have added a new layer (global average pooling (GAP)) to the network structure displayed in Table 1. The performance of the altered network structure is then tested. Figure 10 displays the results of the simulation. See also Figure 11, Figure 12, Figure 13, Figure 14 and Figure 15 and Table 5.

## 7. Conclusions

Spectrum sensing, which differentiates between primary user (PU) activity and its absence, has been viewed as a binary classification problem. A spectrum sensing technique leveraging deep learning principles has been introduced. Using CNN, we suggested many deep learning models to identify whether primary user (PU) activity was present or not. The results of the simulation demonstrated the effectiveness of the suggested approach by outperforming both the frequency domain entropy-based method and the conventional maximum–minimum eigenvalue ratio-based method. Moreover, the method demonstrated robust generalization capabilities, adapting to detect diverse untrained signals. Future work will involve transitioning the model to hardware for performance comparisons with the current model implementation.

## Figures and Tables

**Table 1:** The proposed model architecture.

Layer (Type)	Output Shape	Parameter
Conv. (1D)	(None, 1022, 32)	224
Max pooling (1D)	(None, 511, 32)	0
Conv. (1D)	(None, 509, 64)	6208
Max pooling (1D)	(None, 254, 64)	0
Flatten (Flatten)	(None, 16, 256)	0
Dense (Dense)	(None, 128)	2,080,896
Dropout (Dropout)	(None, 128)	0
Dense_1 (Dense)	(None, 1)	129
Total Parameters		2,087,457
Trainable Parameters		2,087,457
Non-trainable Parameters		0

**Table 2:** RadioML 2018 description.

Parameter	Value or Description
Number of modulation schemes	24
Modulation formats	Digital: GMSK, OOK, 256QAM, 128QAM, 64QAM, 32QAM, 16QAM, 128APSK, 64APSK, 32APSK, 16APSK, 32PSK, 16PSK, 8PSK, 8ASK, 4ASK.
	Analog: FM, AM-DSB-SC, AM-DSB-WC, AM-SSB-SC, AM-SSB-WC.
SNRs (dB)	−20:2:30
Signal format	1/Q format
Vector shape	1024 × 2
Total number of examples	2,555,904
Channel characterization	AWGN
	Doppler shift
	Non-impulsive delay spread
	Symbol rate offset
	Carrier frequency offset
	Selective multipath Rician fading

**Table 3:** The proposed model architecture.

Layer (Type)	Output Shape	Parameter
Input Layer	(None, 1024, 2)	0
Conv. (1D)	(None, 1022, 32)	224
Batch Normalization	(None, 1024, 32)	128
Max pooling (1D)	(None, 512, 32)	0
Dropout (Dropout)	(None, 512, 32)	0
Conv. (1D)	(None, 512, 64)	6208
Batch Normalization	(None, 512, 64)	256
Max pooling (1D)	(None, 256, 64)	0
Dropout (Dropout)	(None, 256, 64)	0
Flatten (Flatten)	(None, 16, 384)	0
Dense (Dense)	(None, 1)	16,385
Total Parameters		23,201
Trainable Parameters		23,009
Non-trainable Parameters		192

**Table 4:** The modified proposed model architecture.

Layer (Type)	Output Shape	Parameter
Conv. (1D)	(None, 1022, 32)	224
Max pooling(1D)	(None, 511, 32)	0
Conv. (1D)	(None, 509, 64)	6208
Max pooling (1D)	(None, 254, 64)	0
Global average pooling (1D)	(None, 64)	0
Dense (Dense)	(None, 128)	8320
Dropout (Dropout)	(None, 128)	0
Dense_1 (Dense)	(None, 1)	
Total Parameters		14,881
Trainable Parameters		14,881
Non-trainable Parameters		0

**Table 5:** Comparison of the proposed model with VGG, AlexNet, and DenesNet models.

Layer (Type)	Output Shape	Parameter
Conv1d_31 (Conv1D)	(None, 1022, 32)	224
Max_pooling1d_9 (MaxPooling 1D)	(None, 511, 32)	0
Conv1d_32 (Conv1D)	(None, 509, 64)	6208
Max_pooling1d_10 (MaxPooling 1D)	(None, 254, 64)	0
Flatten_2 (Flatten)	(None, 16, 256)	0
Dense_7 (Dense)	(None, 128)	2,080,896
Dropout_3 (Dropout)	(None, 128)	0
Dense_8 (Dense)	(None, 1)	129
Total Parameters		2,087,457
Trainable Parameters		2,087,457
Non-trainable Parameters		0

## Data Availability

The original contributions presented in this study are included in the article. Further inquiries can be directed to the corresponding author.
